# Functional Effects of Delivering Human Mesenchymal Stem Cell-Seeded Biological Sutures to an Infarcted Heart

**DOI:** 10.1089/biores.2016.0026

**Published:** 2016-08-01

**Authors:** Katrina J. Hansen, John T. Favreau, Jacques P. Guyette, Ze-Wei Tao, Spencer T. Coffin, Anny Cunha-Gavidia, Brian D'Amore, Luke R. Perreault, John P. Fitzpatrick, Angelica DeMartino, Glenn R. Gaudette

**Affiliations:** ^1^ Department of Biomedical Engineering, Worcester Polytechnic Institute, Worcester, Massachusetts.; ^2^ Center for Regenerative Medicine, Massachusetts General Hospital, Boston, Massachusetts.; ^3^ Department of Pharmaceutical Sciences, University of Arkansas for Medical Sciences, Little Rock, Arkansas.

**Keywords:** biomaterials, cardiology, stem cells, tissue engineering

## Abstract

Stem cell therapy has the potential to improve cardiac function after myocardial infarction (MI); however, existing methods to deliver cells to the myocardium, including intramyocardial injection, suffer from low engraftment rates. In this study, we used a rat model of acute MI to assess the effects of human mesenchymal stem cell (hMSC)-seeded fibrin biological sutures on cardiac function at 1 week after implant. Biological sutures were seeded with quantum dot (Qdot)-loaded hMSCs for 24 h before implantation. At 1 week postinfarct, the heart was imaged to assess mechanical function in the infarct region. Regional parameters assessed were regional stroke work (RSW) and systolic area of contraction (SAC) and global parameters derived from the pressure waveform. MI (*n* = 6) significantly decreased RSW (0.026 ± 0.011) and SAC (0.022 ± 0.015) when compared with sham operation (RSW: 0.141 ± 0.009; SAC: 0.166 ± 0.005, *n* = 6) (*p* < 0.05). The delivery of unseeded biological sutures to the infarcted hearts did not change regional mechanical function compared with the infarcted hearts (RSW: 0.032 ± 0.004, SAC: 0.037 ± 0.008, *n* = 6). The delivery of hMSC-seeded sutures exerted a trend toward increase of regional mechanical function compared with the infarcted heart (RSW: 0.057 ± 0.011; SAC: 0.051 ± 0.014, *n* = 6). Global function showed no significant differences between any group (*p* > 0.05); however, there was a trend toward improved function with the addition of either unseeded or seeded biological suture. Histology demonstrated that Qdot-loaded hMSCs remained present in the infarcted myocardium after 1 week. Analysis of serial sections of Masson's trichrome staining revealed that the greatest infarct size was in the infarct group (7.0% ± 2.2%), where unseeded (3.8% ± 0.6%) and hMSC-seeded (3.7% ± 0.8%) suture groups maintained similar infarct sizes. Furthermore, the remaining suture area was significantly decreased in the unseeded group compared with that in the hMSC-seeded group (*p* < 0.05). This study demonstrated that hMSC-seeded biological sutures are a method to deliver cells to the infarcted myocardium and have treatment potential.

## Introduction

Cardiovascular diseases account for more than 30% of deaths worldwide with myocardial infarction (MI) contributing to about half of these deaths.^1^ Although many advances have been made in revascularization therapy, these therapies do not repair infarcted myocardium.^2,3^

Cell-based therapies have been proposed as a treatment for MI. Importantly for the clinical use of these cells, Hare et al. demonstrated no deleterious effects of human mesenchymal stem cells (hMSCs) when delivered intravenously to patients.^4^ Several clinical trials delivering hMSCs have demonstrated promising initial results with short-term improvements in ejection fraction; longer term studies have shown that functional benefits dissipate within 1 year.^5^ The ability of mesenchymal stem cells (MSCs) to differentiate into contractile myocytes that can couple with the native heart has been debated.^6,7^ However, it appears that this cell type is not able to differentiate into a significant quantity of cardiomyocytes that leads to improved, active contractile function in the myocardium.^8^ Recent studies have suggested that cardioprotective paracrine signaling is largely responsible for the immunomodulatory capacity of MSCs and their ability to improve function after delivery to infarcted tissue.^9,10^

One limitation of MSC therapy is the inefficient engraftment rate using current cell delivery methods.^11^ The most efficient delivery methods are intramyocardial injection, resulting in ∼10% cell retention,^11^ and transendocardial delivery, resulting in ∼19% cell retention.^12^ This small 10–20% retention rate would require a large starting cell population to replace the billion myocytes lost due to an MI.^13^ In addition, targeting cell delivery to a specific region is challenging. To overcome these issues, we developed fibrin-based biological sutures that can be seeded with hMSCs.^14^ Using this delivery method, previous studies have shown a 64% engraftment rate of hMSCs to cardiac tissue.^15^ Herein, we use this delivery method to deliver hMSCs to infarcted myocardium and determine whether these cells lead to improved regional mechanical function.

## Materials and Methods

### Fibrin microthread production

Discrete fibrin microthreads were produced as described previously.^14^ In brief, fibrinogen (70 mg/mL; MP Biomedical) and thrombin (8 U/mL; Sigma) were coextruded in a 10 mM HEPES (Calbiochem)-buffered bath (pH 7.4) using a custom-built system. After extrusion, the microthreads were allowed to polymerize before being removed and air dried.

To create biological sutures, 12 individual microthreads were hydrated with distilled water and twisted together. After drying, each bundle was cut to 4 cm lengths threaded through the eye of a surgical needle (size 26; Havel's, Inc.), hydrated, and twisted together to form a 2 cm long suture. Each suture was placed in a 4 cm long piece of silastic tubing (1.98 mm inner diameter, Dow Corning) with a 27-gauge 0.5 inch needle (Becton Dickinson and Co.) and secured with a slide clamp across the needles. Suture constructs were ethylene oxide sterilized and stored in a desiccator before cell seeding.

### Human MSC culture

Passages 4–8 hMSCs (Lonza, Inc.) were grown in monolayer culture in mesenchymal stem cell growth medium (MSCGM; Lonza, Inc.) incubated at 37°C in 5% CO_2_ until cells reached 80–90% confluence. All implanted hMSCs were treated with MSCGM supplemented with 8.2 nM 655 ITK carboxyl quantum dots (Qdots; Invitrogen) to allow for cell tracking. Qdots were added to 70–80% confluent layers of hMSCs for 24 h, after which Qdot medium was replaced and cells were maintained until further use. Qdot loading was verified using fluorescent microscopy.

### Suture seeding

Before cell seeding, sutures were hydrated with dPBS for 20 min; dPBS is expelled before cells were introduced. For cell seeding, 100 μL of a 2.5 × 10^5^, 5 × 10^5^, and 1 × 10^6^ hMSC/mL solution was drawn into a 1 mL syringe (Becton Dickinson) attached to the 27-gauge needle and dispensed into the tubing containing the suture. The 27-gauge needle and syringe were removed and a slide clamp was added to seal both ends. The seeding construct was placed in a vented 50 mL conical tube and placed in a MACsmix rotator (Miltenyi Biotech) set to four rotations per minute and placed into a 37°C incubator with 5% CO_2_. To determine the role of incubation time, sutures were incubated for 1, 3, 6, 12, and 24 h. All implanted sutures were seeded with 100 μL of a 1 × 10^6^ hMSC/mL solution for 24 h.

### hMSC attachment quantification

After seeding, hMSC-seeded sutures not destined for implantation were either fixed in 4% paraformaldehyde for staining or prepped for a CyQuant DNA assay. For CyQuant DNA assays, sutures were placed in a 1.5 mL Eppendorf tube with 100 μL of phosphate-buffered saline (PBS) in a −80°C freezer for cell lysis. The CyQuant assay was run according to manufacturer's specifications. Fluorescent measurements were taken using a plate reader (Victor 3; Perkin Elmer) at an excitation of 480 nm and absorption of 520 nm. Values are reported as mean ± standard error of the mean (SEM) cell count per linear centimeter of suture.

### Infarct model

**FIG. 1.**
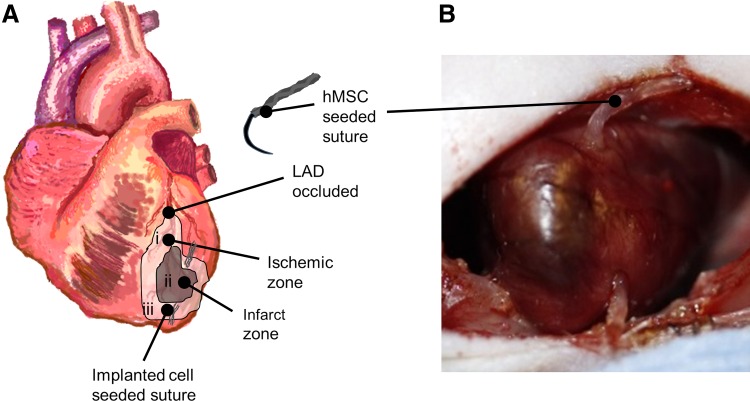
Implanted hMSC-seeded suture. Male athymic rat heart was exposed and the LAD was ligated to induce infarct. One hour postligation, ligature was cut and blood flow was resumed. The hMSC-seeded fibrin suture was implanted from the base to the apex such that suture entered in the ischemic zone (i), passed through the infarcted myocardium (ii), and exited through the ischemic zone at the apex (iii) **(A)**. Implantation of fibrin suture in rat heart **(B)**. Excess suture was removed. hMSC, human mesenchymal stem cell; LAD, left anterior descending.

Male nude rats (NIH nude; Taconic) were used for all procedures. Animals were anesthetized using 5% inhaled isoflurane in oxygen, intubated, and mechanically ventilated with anesthesia maintained with 3% isoflurane. A 2 cm incision along the fourth intercostal space and subsequent blunt dissection of the muscle was used to expose the heart. To induce an infarct, the left anterior descending (LAD) artery was ligated for 1 h using 5-0 Prolene (Ethicon) suture. After 1 h, the ligature was cut to restore blood flow and the chest was either closed or the suture was implanted and the chest was closed. The suture was implanted from the base of the heart toward the apex of the heart, and excess suture was cut and removed (Fig. 1). Sham operations underwent the same procedure already described without the ligation of the LAD and suture implantation. Animals were allowed to recover for 1 week.

### Terminal surgery

One week postoperation, animals were anesthetized, intubated, and placed on a respirator as already described. The heart was exposed through the original suture lines and retractors were placed into the intercostal space to expose the left ventricle of the heart. A 3.5 French size micro tip catheter (Millar) was inserted into the left ventricle through the apex and sutured in place. A high speed camera (Fastec HiSpec4; Fastec, Inc.) was used to record high speed videos (250 frames/sec, 8 bit depth, 1696 × 1710) of contracting ventricular muscle. Pressure data were synchronized to video frame acquisition through a data acquisition board (National Instruments) and a LabVIEW-based controller. Videos were acquired from multiple angles and video and pressure data were saved for offline data analysis. After video acquisition, a 0.3 mg/kg dose of Beuthanasia-D (VetOne) was injected into the left atrium to arrest the heart, and heart tissue was removed for histological processing and analysis.

### Assessment of global and regional mechanical function

Global mechanical function was assessed using the collected pressure waveforms as described previously.^16^ Maximum developed pressure was determined by averaging the difference between the maximum and minimum pressures for each beat. Minimum rate of pressure decline and maximum rate of pressure development were determined by applying a five-point linear averaging filter to the pressure wave form and finding the maximum and minimum of the discrete derivative of the signal for each beat. Finally, the diastolic relaxation time constant was determined by applying a three-parameter least squared fitting method to pressure data from its negative inflection point to its minimum for each beat.^17^ All global function data are averaged over at least five beats per animal.

High density mapping (HDM) was used to assess regional mechanical function as previously described.^15,16,18^ HDM is a speckle tracking algorithm based on Fourier domain phase correlation with the subpixel registration algorithm described by Foroosh et al.^19^ Using this method, a region of interest was defined on the rat heart over the thread and/or infarct region. The region was subsequently divided into small (32 × 32 pixel) windows. Individual windows were tracked from frame to frame across several cardiac cycles to yield a displacement field. Displacement fields were used to quantify systolic area of contraction (SAC) and regional stroke work (RSW), as described previously.^18^

### Histology

Sutures not prepared for CyQuant were fixed in 4% paraformaldehyde for 10 min and stained for F-actin. In brief, sutures were washed in PBS, blocked for 30 min in 1% albumin from bovine serum, and stained with 488 phalloidin (Invitrogen) for 30 min and counterstained with Hoechst 33342 (0.5 μg/mL; Invitrogen) for 5 min at room temperature.

After terminal surgery, hearts were removed and cut into half bisecting the infarct and biological suture, and fixed in 4% paraformaldehyde overnight. After fixation, heart sections were moved to a 30% sucrose solution for 24 h. Each half was embedded using OCT and placed in a Leica CM 3050 cryostat (Leica Microsystems) and cut into 10 μm thick sections onto charged glass microscope slides (Globe Scientific, Inc.). Sections were stained with Masson's trichrome reagents according to manufacturer's instruction. Adjacent sections were used for immunofluorescence staining. In brief, sections were fixed in acetone at −20°C for 10 min and blocked with 5% goat serum for 45 min. The sections were incubated in mouse anti-α-actinin monoclonal antibody (1:100; Abcam) overnight at 4°C. Subsequently, sections were treated in goat antimouse secondary antibody (1:400; Alexa Fluor 488; Thermo Fisher Scientific) 1 h at room temperature and counterstained with Hoechst 33342 (0.5 μg/mL) for 5 min at room temperature. Images were acquired with a Leica Upright DMLB2 or a Leica TCP SP5 confocal laser scanning microscope.

### Infarct region and suture area quantification

Microsoft Image Composite Editor was used to generate composite images of the full cross sections from sections stained with Masson's trichrome. Images were uploaded into a custom MATLAB analysis program (Mathworks) that evaluates color of individual pixels in the image and extrapolates the location of the lumen and outer boundary of the section. Image regions outside the boundary of the heart and within the lumen (white space) were removed, leaving the image region corresponding to intact heart tissue. As Masson's trichrome stains cardiac muscle tissue red and fibrosis blue, blue regions of the image were programmatically identified and used to determine infarct size. The program approximates and outputs total section area and total area of the infarct. Sectional and infarct area was calculated by the sum of squared pixel lengths and converted to microns to determine the percentage of the section that is infarcted.

For suture area measurement, images with Masson's trichrome staining were uploaded to ImageJ (NIH). Manual tracings around the suture in each cross section were obtained to give the area of the suture. Every section containing a suture was used for the measurements to give an average area.

### Statistics

Results are represented as mean ± SEM. Comparisons between seeding conditions were analyzed using a one-way analysis of variance, with a *post hoc* Tukey test for multiple comparisons. Comparisons between *in vivo* treatment conditions were analyzed using a Kruskal–Wallis test with a Dunn's test for multiple comparisons. An unpaired *t* test was used to determine significance between suture areas. All conditions were considered significant at a value of *p* < 0.05.

## Results

### Concentration and duration of cell seeding affect cell attachment to biological sutures

**FIG. 2.**
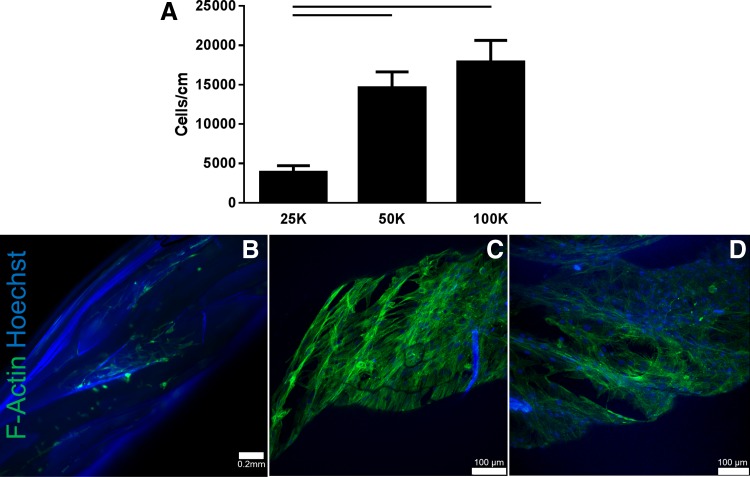
Effect of cell seeding concentration on hMSC attachment to biological suture. Biological sutures were seeded with three different concentrations of hMSCs: 25, 50, and 100 k cells per suture. Highest attachment was found for the higher concentrations **(A)** with no statistical difference found between the 50 and 100 k. F-actin staining for 25 k **(B)**, 50 k **(C)**, and 100 k **(D)** revealed similar cell attachment for the 50 and 100 k conditions. Some blue autofluorescence seen in **(B)** and **(D)** is due to the suture. Mean ± SEM, *p* < 0.05, *n* = 10 per group. SEM, standard error of the mean.

Three concentrations of hMSCs (2.5 × 10^5^, 5 × 10^5^, and 1 × 10^6^ hMSCs/mL solution) were seeded onto 2 cm long biological sutures. There were significantly more hMSCs attached to the sutures seeded with the higher concentrations (5 × 10^5^ and 1 × 10^6^ hMSC/mL) than a concentration of 2.5 × 10^5^ (*n* = 10; *p* < 0.05) (Fig. 2A), a finding that was further supported by F-actin staining (Fig. 2B–D).

**FIG. 3.**
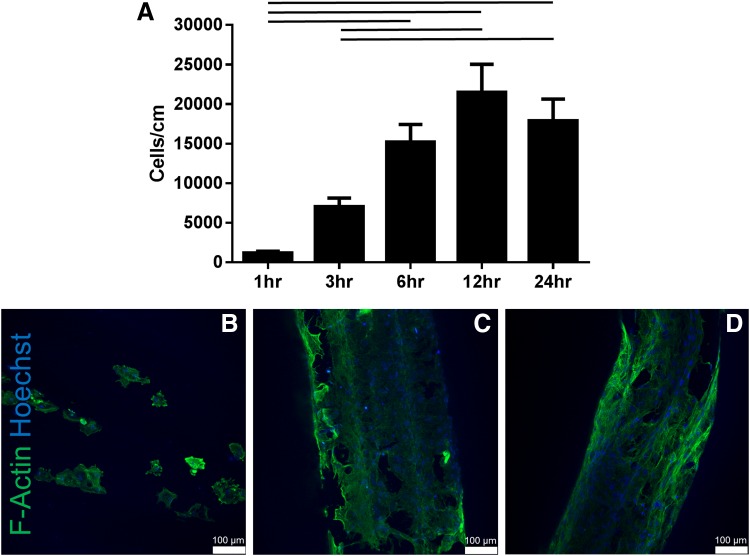
Effect of seeding time on hMSC attachment to biological suture. hMSC attachment for five different time points (1, 3, 6, 12, and 24 h) was examined. Seeding times of 1 and 3 h resulted in significantly fewer cells attached compared with seeding times 12 and 24 h, where there was no significant differences in attachment at 6, 12, and 24 h **(A)**. F-actin staining shows cells attached at 1 h **(B)**, but with higher number of cells attached at 6 and 12 h **(C, D)**. Mean ± SEM, *p* < 0.05, *n* = 10 per group.

To determine the effect of incubation time, five different times were examined (1, 3, 6, 12, and 24 h). Seeding for 1 h (1216 ± 192, *n* = 10) and for 3 h (7052 ± 1085, *n* = 10) significantly reduced cell attachment compared with seeding for 12 h (21,512 ± 3526, *n* = 10) and 24 h (17,887 ± 2746, *n* = 10) (*p* < 0.05) (Fig. 3A). There was no significant difference in the number of hMSCs attached at 6 (15,224 ± 2213, *n* = 10), 12, or 24 h. F-actin staining shows cell attachment beginning at 1 h (Fig. 3B), more cells attached at 6 and 12 h (Fig. 3C, D), with cells appearing to elongate along the length of the suture at 12 and 24 h.

### hMSC-seeded biological sutures do not improve global function after infarction

After the model was successfully developed, five sham, two infarct, one unseeded, and three animals from the hMSC-seeded group died before successful completion of all data acquisition. These animals were not included in this study.

**FIG. 4.**
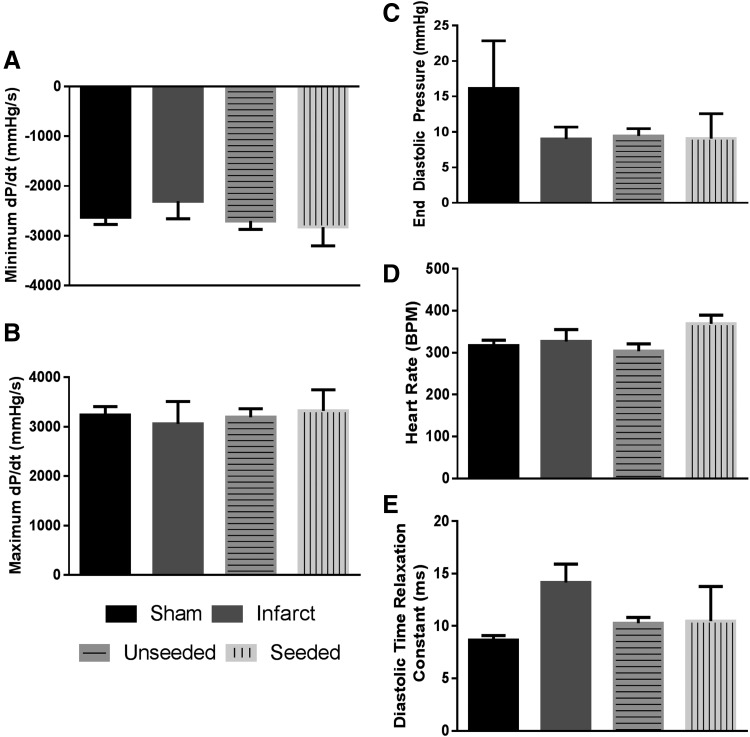
Global function is not impacted by hMSC-seeded biological suture treatment after infarct. No significant changes were noted for any of the following measurements: minimum dP/dt **(A)**, maximum dP/dt **(B)**, end diastolic pressure **(C)**, heart rate **(D)**, or diastolic time relaxation constant **(E)**. Mean ± SEM. *n* = 6 per group.

No differences were noted in the maximum or minimum rate of pressure development or end diastolic pressures between groups (sham, MI, MI with unseeded sutures, and MI with hMSC-seeded sutures, *n* = 6 per group; Fig. 4A–C). There were no significant differences in the heart rates in all groups at the time of terminal surgery (Fig. 4D). Similarly, no significant differences were seen in diastolic relaxation time constant, although sham-operated hearts had the shortest relaxation time (8.6 ± 1.1 msec) and infarcted hearts had the longest relaxation time (14.1 ± 4.3 msec) (Fig. 4E).

### hMSC-seeded biological sutures improve regional mechanical function after infarction

**FIG. 5.**
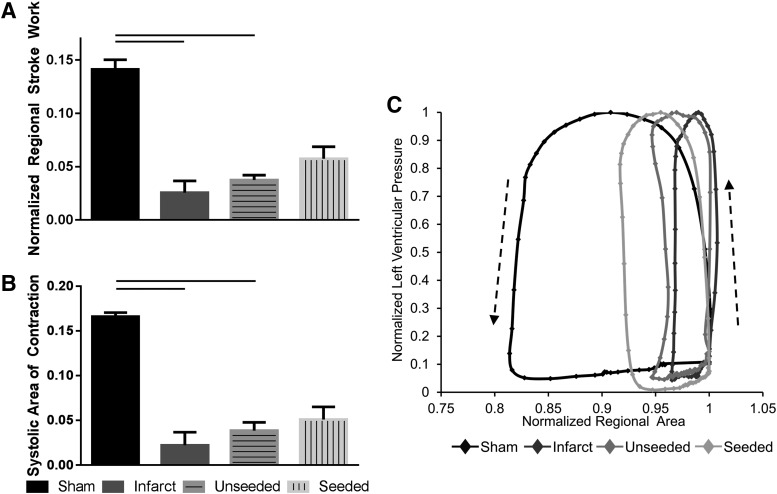
hMSC-seeded biological sutures improve regional mechanical function after infarct. RSW and SAC were significantly decreased for infarct and unseeded suture groups compared with the sham group **(A, B)**. Representative pressure area work loop **(C)** shows improved function for seeded group compared with infarct and unseeded groups. Arrows indicate direction of all work loops over time. Mean ± SEM, *p* < 0.05, *n* = 6 per group. RSW, regional stroke work; SAC, systolic area of contraction.

To assess regional mechanical function, a speckle tracking algorithm, HDM, was used for determination of RSW and SAC. The average region in which function was determined was similar in all groups. Creation of an MI resulted in a significant decrease in RSW (0.026 ± 0.011) and SAC (0.022 ± 0.015) compared with sham-operated animals (RSW 0.141 ± 0.009; SAC 0.166 ± 0.005) (*n* = 6, *p* = 0.0027 RSW and SAC) (Fig. 5A, B). Delivery of unseeded sutures to the infarcted regions also resulted in statistically reduced RSW (0.037 ± 0.005) and SAC (0.039 ± 0.009) compared with sham-operated animals (*n* = 6, *p* = 0.012 RSW; *p* = 0.026 SAC). hMSC-seeded sutures had a lower RSW (0.057 ± 0.011) and SAC (0.051 ± 0.014) than sham-operated hearts, although statistical significance was not achieved (*n* = 6, *p* = 0.165 RSW; *p* = 0.086 SAC). There were no significant differences in regional mechanical function between infarct, unseeded, and seeded groups; however, infarcted hearts treated with cell-seeded sutures showed a trend toward increase of RSW when compared with infarcted hearts (*p* = 0.07). Representative pressure area work loops (Fig. 5C) indicate improved function in hMSC-seeded group over unseeded and infarcted groups.

### hMSCs delivered on biological sutures remain in heart at least 1 week after delivery

**FIG. 6.**
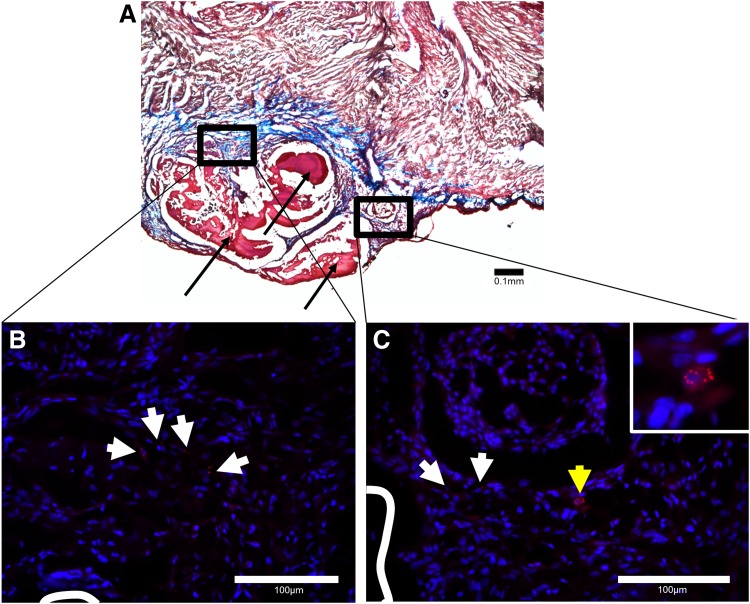
hMSCs persist in myocardium 1 week after delivery. Masson's trichrome-stained cross sections show successful delivery of biological suture [pink; black arrows in **(A)**, white lines in **(B)** and **(C)**] to myocardium. The black boxes **(B, C)** show the location of serial sections stained for α-actinin and Hoechst relative to **(A)**. The arrows in **(B)** and **(C)** indicate quantum dot-positive hMSCs that have migrated off the suture into fibrotic tissue. The cell identified by the yellow arrow in **(C)** is shown in the inset.

Numerous Qdot-loaded cells were detected in the seeded group. Cells were found in regions close to the biological sutures (Fig. 6), but not on the suture, suggesting migration into surrounding tissue. When stained to observe α-actinin, no Qdot-loaded cells observed exhibited positive α-actinin expression.

### Effects of implantation of unseeded and hMSC-seeded sutures on infarct size

**FIG. 7.**
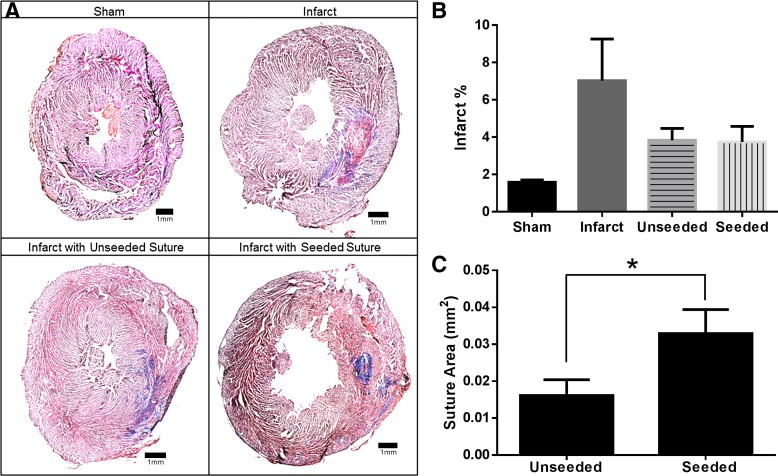
Infarct size and suture area. Masson's trichrome staining shows infarcted area in blue and healthy muscle in red **(A)**. Quantification of infarct size from serial trichrome-stained sections indicates an increase in infarct size for infarct-only group with a similar reduction in infarct size shown for seeded and unseeded groups (*n* = 6) **(B)**. Quantification of persisting suture area after 1 week shows significantly smaller suture area for unseeded sutures **(C)**. Mean ± SEM, *p < 0.05.

Serial sections of Masson's trichrome-stained sections were analyzed to determine infarct size, denoted as a percentage of the cross section. No infarcted tissue was detected by Masson's trichrome staining in sham-operated hearts, whereas the MI, unseeded suture, and seeded suture groups all appeared to have infarct regions (Fig. 7A). Infarcted hearts without suture delivery had the greatest infarct size of 7.0% ± 2.2% (Fig. 7B). Unseeded and hMSC-seeded groups had comparable infarct sizes of 3.8% ± 0.6% and 3.7% ± 0.8%, respectively, but were not significantly different from MI alone (*p* > 0.05, Fig. 7B). Due to the automated method used to calculate infarct size, the sham group had a small infarct size of 1.6% ± 0.1% (likely due to inherent collagen structure in the healthy heart).

### hMSCs preserve suture area in infarct

Serial sections of Masson's trichrome-stained sections were analyzed for suture area. An average suture area of 0.016 ± 0.004 mm^2^ and 0.033 ± 0.007 mm^2^ was found for unseeded and seeded groups, respectively (Fig. 7C). The group with hMSCs had a significantly greater suture area (*p* < 0.05), indicating that the hMSCs preserved suture area at 1 week after delivery in the infarcted heart.

## Discussion

Cell therapy continues to hold tremendous potential to treat millions of patients living with MIs and heart failure. However, the full benefits of cell therapy have yet to be realized. The past decade yielded dozens of clinical trials aimed at improving cardiac output by examining different cell types, including bone marrow mononuclear cells,^20^ MSCs,^4,21^ and cardiosphere-derived cells,^22^ effect of timing on cell delivery,^23–25^ chronic^26,27^ and acute^28^ MI, and cell delivery methods: intracoronary,^22,28^ intravenous,^4^ intramyocardial,^29^ and transendocardial.^12,30^ Despite numerous trials investigating extensive variables, these clinical trials demonstrated limited improvements in ejection fraction without restoring cardiovascular function to baseline values. All studies failed to efficiently deliver the chosen cell type to infarcted tissue, further underscoring the value of investigating new delivery methods that improve cell retention.

Current cell delivery methods to the heart exhibit retention rates of only 10–20%, which are inefficient for delivering clinically relevant number of cells.^11–13^ Previously, we demonstrated cell-seeded biological sutures capable of efficiently delivering cells to a targeted area (64% retention).^15^ In addition, we reported that delivering unseeded sutures to a healthy heart demonstrated a small fibrotic reaction that was significantly reduced with hMSC-seeded sutures.^16^ In terms of regional function, unseeded sutures decreased SAC, where hMSC-seeded sutures dampened the decrease in SAC.^16^ Similar values for RSW and SAC were reported for sham animals in the previous study and in our current study. In this study, we investigated the functional effects of delivering hMSCs through a biological suture to an infarcted heart. When combined with our method for evaluating regional mechanical function with high spatial resolution, we are uniquely able to determine changes in regional mechanical function that occur directly in the region of cell delivery. This combination allows for targeted cell delivery followed by targeted evaluation of regional mechanical function.

In this study, we demonstrated that varying cell seeding concentrations and seeding times affect the quantity of hMSCs seeded onto the suture. Ideal seeding conditions were found to occur using 100,000 cells per suture seeded for 12–24 h. There was only a small increase in cell attachment between the 50,000 and 100,000 seeded groups, thus we do not believe that further increasing the number of cells seeded would lead to improved cell attachment as the surface area available for cell attachment has been maximized. Due to time considerations for animal studies, sutures seeded for 24 h with 100,000 cells were used (allowing for seeding 24 h before implantation).

No changes between conditions were found for global parameters including pressure changes, diastolic relaxation constant, or heart rate. The MI created in the rat may not have been large enough to result in a detectable change in global function. In addition, rat ventricular remodeling after an MI can take 4–8 weeks to manifest such that changes on a global level may not be seen at 1 week.^31^ This finding further necessitates the use of HDM, which images the region of damage and cell delivery to determine on a regional level whether improvements in mechanical function were found that may have been negated at a global level.

Using HDM to analyze myocardial function in infarcted rat hearts we demonstrated decreased regional mechanical function in animals with an induced MI, using metrics of RSW and SAC. Delivering unseeded sutures demonstrated improvements in regional mechanical function over the MI group; the hMSC-seeded sutures demonstrated slightly higher function, however, not statistically significant. This suggests that the biological suture alone has the ability to improve regional mechanical function in the infarct zone. Studies have demonstrated that acellular biomaterial delivery to an infarct can improve mechanical function by stabilizing the infarct, thickening the left ventricle, and decreasing infarct expansion.^32–34^ As both the unseeded and hMSC-seeded suture groups had similar infarct sizes, the significantly increased suture area for the hMSC-seeded group may explain the improvement in regional mechanical function for the hMSC-seeded group compared with the unseeded group. A similar trend of increased suture area for hMSC-seeded sutures was also found in studies in a healthy heart, suggesting the ability for the hMSCs to decrease *in vivo* degradation time of biological sutures compared with unseeded sutures.^16^

It has been established that MSCs act transiently and die off within days to weeks of being delivered to the infarct.^8^ As such, we chose not to examine cell retention as hMSC retention may be altered by the infarct environment at 1 week independent of the method used for delivery. However, hMSCs were found to persist in the myocardium for 1 week and were found in regions away from the suture, suggesting their propensity to migrate from the suture into the damaged tissue. Delivered cells were tracked using Qdots, which have previously been shown to be taken up by hMSCs and remain in the cells for several passages *in vitro*.^35^

Despite the emergence of many clinical trials focused on the delivery of MSCs to injured cardiac tissue, none of these trials have demonstrated significant findings to suggest the robust ability for MSCs to improve contractile cardiac function. Many of these studies used delivery methods with low delivery efficiencies. In this study, we describe findings using a biological suture-based method to deliver cells to infarcted tissue. Although we did not see statistically improved function when seeded sutures were delivered over infarct alone, we do remain optimistic about the use of the biological suture for cell delivery. Although hMSCs may not be the most appropriate cell to use in regeneration of contractile cardiomyocytes, they may still have beneficial effects in the infarcted heart as studies have demonstrated their immunomodulatory effect.^36,37^ Recent advances in induced pluripotent and embryonic stem cell technology have enabled the development of a cardiomyocyte that has demonstrated the *in vitro* contractile properties that may be ideal for cell replacement strategies.^38,39^ Future studies will focus on delivering stem cell-differentiated cardiomyocytes using our biological suture technology.

## Abbreviations Used

HDM: high density mapping
hMSC: human mesenchymal stem cell
LAD: left anterior descending
MI: myocardial infarction
MSC: mesenchymal stem cell
MSCGM: mesenchymal stem cell growth medium
PBS: phosphate-buffered saline
Qdot: quantum dot
RSW: regional stroke work
SAC: systolic area of contraction
SEM: standard error of the mean
